# Distinct features of EEG microstates in autism spectrum disorder revealed by meta-analysis: the contribution of individual age to heterogeneity across studies

**DOI:** 10.3389/fpsyt.2025.1531694

**Published:** 2025-04-22

**Authors:** Ran Wei, Yonglu Wang, Hui Fang, Luyang Guan, Jianxing Gao, Xinyue Xu, Xiaoyan Ke, Hua Jin

**Affiliations:** ^1^ Children's Mental Health Research Center of the Affiliated Brain Hospital of Nanjing Medical University, Nanjing, China; ^2^ Child Healthcare Department of the Affiliated Suzhou Hospital of Nanjing Medical University, Suzhou, China; ^3^ Child Healthcare Department of the Suzhou Maternal and Child Health Hospital, Suzhou, China

**Keywords:** autism spectrum disorder, age-dependence, EEG microstate, meta-analysis, EEG

## Abstract

**Background and purpose:**

Electroencephalographic (EEG) microstates, as quasi-stable scalp EEG spatial patterns, are characterized by their high temporal resolution, making them a potentially powerful approach for studying the function of large-scale brain networks. A substantial body of research has demonstrated that abnormalities in the function or structure of large-scale brain networks are closely related to many characteristics of autism spectrum disorder (ASD). Investigating the EEG microstate features of individuals with autism can help reveal the nature of autism. To date, numerous studies have observed unique resting-state microstate patterns in individuals with autism. However, the results of these studies have not been consistent. Therefore, the present study aims to assess the differences in microstate parameters between ASD and non-autistic groups through meta-analysis and to explore the sources of research heterogeneity.

**Method:**

This meta-analysis was preregistered with PROSPERO (CRD42024599897) and followed PRISMA guidelines. Studies in English comparing EEG microstate patterns between ASD and Non-autistic groups were retrieved by database search to October 20, 2024. The meta-analysis was then conducted using RevMan5.2. Pooled results are expressed as standardized mean difference (SMD). Heterogeneity (I²) and publication bias were assessed using Stata15.0.

**Result:**

Seven studies enrolling 194 ASD individuals were included, four deemed high quality and three moderate quality according to bias risk assessment. Microstate B duration and coverage were significantly greater in the pooled ASD group (duration SMD=0.83, 95%CI: 0.17–1.5; coverage SMD=0.54, 95%CI: 0.18–0.90), but heterogeneity could not be excluded. Microstate C occurrence frequency was also in the ASD group (SMD= -0.61, 95%CI: -1.08 to -0.15), and heterogeneity was significant. Sensitivity analysis revealed that only the group difference in microstate B coverage was robust. Subgroup analysis suggested that age was the main source of heterogeneity in microstate B and C coverage. Results were not affected by publication bias according to Egger’s test.

**Conclusion:**

Future studies on the EEG microstate characteristics of ASD must control for age as an important cofounding variable.

**Systematic Review Registration:**

PROSPERO, identifier CRD42024599897

## 1 Background

Autism spectrum disorder (ASD) is a neurodevelopmental condition characterized by differences in social communication, focused interests, and repetitive behaviors (1, 2). The global median prevalence of ASD is about 100 cases per 10,000 people, or approximately 0.6% of the general population, and is currently on the rise (3, 4). The core characteristics of ASD can substantially impede academic achievement, career success, and social functioning. Moreover, autism is associated with greater risks of epilepsy and sleep disorders (5–7). Neuroimaging studies, including functional magnetic resonance imaging (fMRI) investigations, have revealed Large-scale brain networks level abnormalities in ASD that may constitute the neurophysiological bases for the condition occurrence and development (8–12).

A brain network study (13) involving 152 individuals with ASD and 159 healthy comparisons, based on the Autism Brain Imaging Data Exchange (ABIDE) database, revealed that ASD participants exhibited lower energy levels in the default mode network (DMN) and salience network (SN) compared to healthy comparisons. This finding suggests a lack of dynamic switching and flexibility within the brain network, which may be associated with the core characteristics of ASD. Another study (14) utilizing the ABIDE database investigated changes in the DMN subsystems of ASD individuals during childhood and adolescence. The results indicated that the connection strength between DMN subsystems decreased in the ASD group, while the DMN subsystem organization remained relatively stable in the comparison group. This suggests that the development of the DMN in ASD individuals may be delayed. A large-scale meta-analysis (15) of 1728 individuals with ASD and 1747 typically developing (TD) individuals also demonstrated structural and functional abnormalities in the DMN. Specifically, increased spontaneous activity was observed in the right precuneus, while decreased functional activity was noted in the right inferior temporal gyrus (ITG) and left angular gyrus. These abnormalities are likely closely related to core characteristics such as differences in social communication. Collectively, these findings suggest that structural, functional, and developmental abnormalities in large-scale brain networks may underlie the pathogenesis of ASD.

However, fMRI has limited temporal resolution, so abnormalities in highly dynamic neural processes may be missed. In contrast, EEG can record brain electrical activity with millisecond temporal resolution, complementing fMRI for assessment of ASD-associated network dysfunction (16, 17).

As a novel method for analyzing EEG signals, an EEG microstate is a brief period of quasi-stable scalp potential topography in EEG recordings, characterized by unique spatial patterns that last for about 60-120 milliseconds before transitioning to another state, reflecting millisecond level large-scale brain network activity with its high temporal resolution. EEG microstates are believed to reflect rapidly changing neural activity (18).

Microstates are topologically represented by template maps derived from EEG signals through spatial clustering analysis methods, such as k-means clustering and the topological atomization and aggregation hierarchical clustering (T-AAHC) algorithm (19), These methods identify multiple distinct microstates, with four patterns emerging as the most consistent across studies. These patterns exhibit spatially opposite polarity characteristics on topographic maps: Microstate A (right frontal–left posterior), Microstate B (left frontal–right posterior), Microstate C (anterior–posterior), and Microstate D (central–peripheral) (18, 20, 21). Overall, these Microstate account for 65% to 85% of the total terrain variance recorded by EEG (18, 22). In terms of function, Britz et al. (21), Yuan et al. (46), Musso et al. (45) have suggested that microstate A is associated with the phonological processing network after investigating the relationship between the blood oxygen level-dependent (BOLD) signal in functional magnetic resonance imaging and the parameters of EEG microstates. Microstate B is related to the visual network. Microstate C is associated with the salience network. Microstate D is related to the attention network.

The typical time parameters calculated for microstates include the following: (1) the average duration that a given microstate remains stable, (2) the frequency of occurrence for each microstate, which is independent of its individual duration, and (3) the proportion of the total recording time dominated by a given microstate, also referred to as its fraction or coverage (18, 20). And there is mounting evidence that these parameters are abnormal in neuropsychiatric and neurodevelopmental disorders, including schizophrenia and mood disorders (22–25). Moreover, microstate pattern was also reported to reflect characteristic changes follow repetitive transcranial magnetic stimulation (rTMS) (26, 27). Therefore, the characterization of EEG microstate abnormalities may prove useful for the condition diagnosis, prognosis, and treatment evaluation.

Recent studies have investigated the characteristics of EEG microstates in individuals with ASD. For instance, D’Croz Baron et al. (28) and Bochet et al. (29) reported that ASD group exhibited longer average duration, higher frequency, and greater time coverage of microstate B compared to comparisons. Jia et al. (30) observed that the frequency and time coverage of microstate B were elevated in the ASD group, while the duration and time coverage of microstate C decreased. Additionally, they found reduced duration of microstate A and increased frequency of microstate D. Nagabushhan et al. (31) noted increased frequency and coverage of microstate B, as well as longer duration and higher frequency of microstate C. takarae et al. (32) identified an increased occurrence frequency of microstate C. In a recent study (33), ASD group showed decreased duration, frequency, and coverage of microstate C, with no significant changes in these metrics for microstate B. However, the time coverage of microstate A was found to increase. Iftimovici et al. (25) reported significantly reduced frequency and time coverage of microstate D in the ASD group. Despite these findings, significant heterogeneity across studies has been observed, likely attributable to differences in subject age, microstate clustering methods, and/or sample size.

Das et al. (34) recently summarized research findings on the distinct EEG microstate characteristics of ASD as of May 30, 2022, and concluded that the time parameters of microstates B and C often differed between ASD and TD groups. Further, they also analyzed the reasons for differences in results across studies but did not provide a quantitative analysis or delve into the origins of the observed heterogeneity. Moreover, subsequent to publication, a number of new EEG microstate studies have appeared (25, 33). Consequently, the objective of the current study is to conduct a quantitative synthesis of the existing research, including the most recent studies, to identify specific microstate parameters that distinguish ASD from TD. By assessing the robustness of these differences, examining potential publication biases, and quantifying the sources of heterogeneity across studies, we also aim to provide guidance for future studies on the EEG microstate characteristics of ASD.

## 2 Methods

This meta-analysis was registered in advance with PROSPERO (https://www.crd.york.ac.uk/prospero) (CRD42024599897) and written in accordance with the 2020 Preferred Reporting Items for Systematic Reviews and Meta-analyses (PRISMA) guidelines (35). The PRISMA checklist can be found in **Supplementary Table 1**.

### 2.1 Inclusion criteria

The following criteria were preset for literature inclusion according to the purpose of this meta-analysis: (a) articles written in English, (b) including participants of any age (children, adolescents, or adults), (c) at least four microstate types identified and analyzed (A, B, C, and D), (d) comparing an ASD group to a matched TD group, (e) analyses based on resting EEG, (f) sufficient data available for calculation of SMD, I^2^, and effective size, and (g) ASD diagnosed according to accepted guidelines (DSM- IV/V or ICD-10). Exclusion criteria are as follows: (a) non-English literature, (b) fewer than four microstates identified, (c) no comparison group, (d) event-related potential (ERP) or other non-resting EEG study, (e) insufficient data to calculate SMD, I^2^, and effective size, and (f) enrolling individuals with other neurodevelopmental disorders such as attention deficit hyperactivity disorder (ADHD), mental retardation, and tic disorder.

### 2.2 Information sources and searches

We conducted searches of PubMed, Web of Science, EMBASE, EBSCO, PsychoInfo, and Cochrane Library, with a deadline of October 20, 2024, and literature search was conducted without restrictions on document type, encompassing studies, reviews, case reports, and clinical trials. The search strategy included keywords related to (a) ASD, (b) EEG, and (c) microstates. The specific search strings are detailed in **Supplementary Table 2**.

### 2.3 Study selection

The three authors (WR, WYL, GLY) identified potentially eligible articles meeting inclusion criteria. After removing duplicates, two independent reviewers (WR, WYL) screened all potentially eligible articles according to title and abstract contents. Finally, full texts of the remaining potentially eligible articles were reviewed and authors reached a consensus based on inclusion criteria. Ultimately, seven articles were selected for inclusion.

### 2.4 Outcome measures

The primary outcome measures were duration, frequency of occurrence, and coverage of four microstates (A, B, C, D), in accord with previous studies on other psychiatric disorders (36).

### 2.5 Data extraction

Two authors (GJX, XXY) downloaded the bibliography and used EndNote for unified bibliography management. The following study parameters were extracted independently for ASD and TD groups and recorded in Office Excel: number of participants, number of males (%), mean age, whether EEG data were collected with eyes open or closed, and the duration, frequency of occurrence, and coverage of each EEG microstate. In addition, the microstate algorithm (k-means or T-AAHC) was recorded for each included study. We contacted the authors of included studies to collect any missing data, and studies were excluded if no data were provided. Alternatively, studies remained in the analyses if some data (e.g., a missing parameter) were provided (30, 32).

### 2.6 Risk of bias and quality assessment

All studies ultimately included in this meta-analysis were cross-sectional, so methodological quality was rated by three authors (FH, JH, WR) using an evaluation form provided by the Agency for Healthcare Research and Quality (AHRQ) for assessment of cross-sectional studies (37), and any differences were resolved through discussion. The AHRQ form has 11 items, each answered ‘yes’ (1 point) or ‘no’ (0 points) for a total score from 0 to 11. A total score of 0 to 3 points is deemed low quality, 4 to 7 points as moderate quality, and 8 to 11 points as high quality.

### 2.7 Meta-analysis

The meta-analysis of outcome variables was conducted using RevMan version 5.2 (38) according to the methods recommended in the Cochrane Handbook (JPT and Editors 2008). All major outcomes are continuous variables, and so each is expressed as the standardized mean difference (SMD) between groups with 95% confidence interval, while study heterogeneity is expressed by *I*² *(* 39). In the absence of significant heterogeneity, the data were analyzed using a fixed effects model, while pooled data with heterogeneity across source studies were analyzed using a random effects model. Subgroup analyses stratified by age and microstate clustering algorithm were conducted to assess the impacts of these possible sources of heterogeneity.

For evaluation of stability and robustness, we conducted stability analysis using the Leave-One-Out method (40), as applied by the Metan module of Stata15.0. Finally, publication bias was evaluated using the Egger’s test function of Stata 15.0 with a two-tailed P < 0.05 considered statistically significant (41).

## 3 Results

### 3.1 Search results

**Figure 1:**
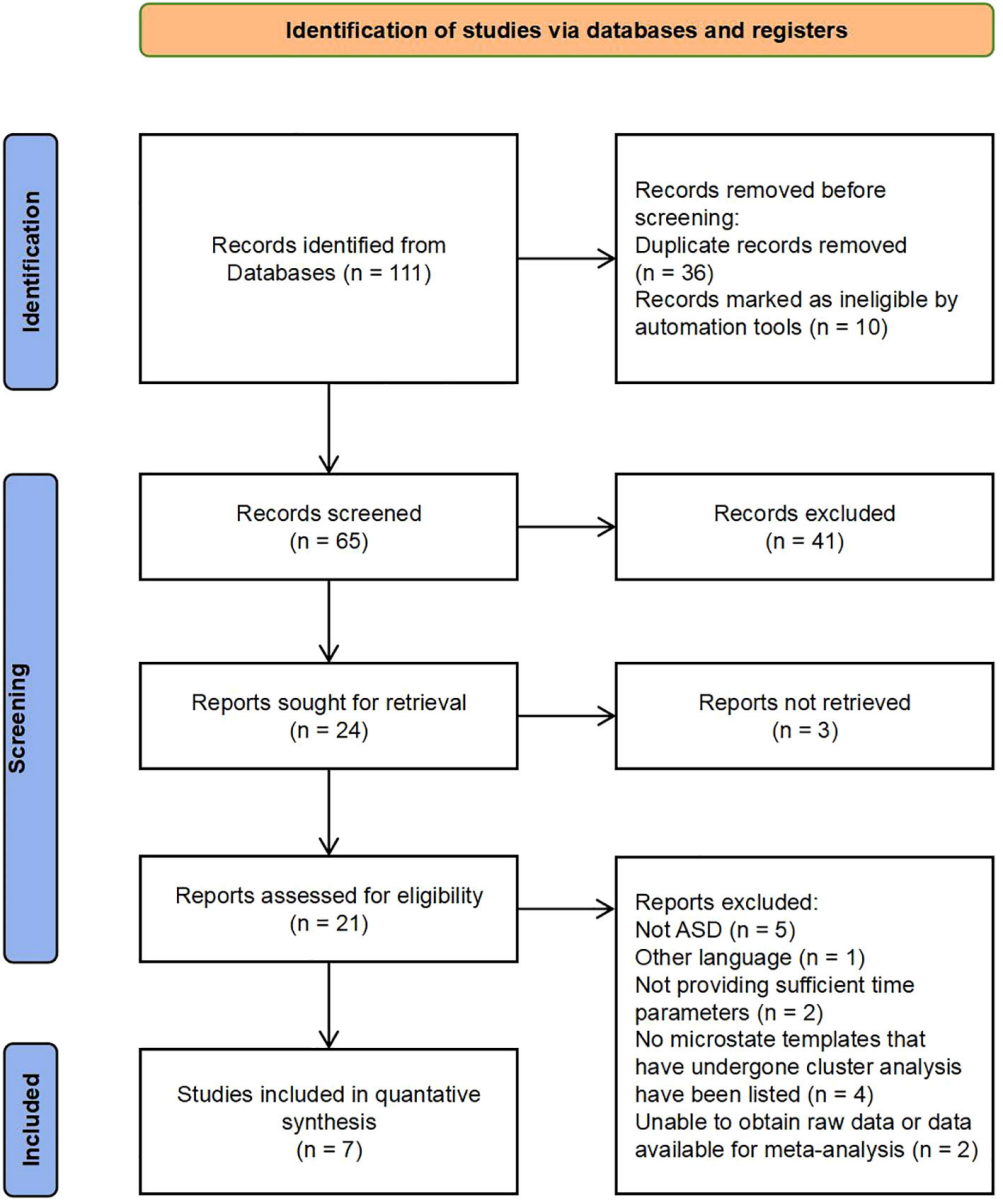
PRISMA flowchart (adapted from (35).

A total of 111 articles were retrieved using the search item strings presented in **Supplementary Table 2**. Duplicates (n = 36) and incomplete entries (n = 10) were then removed using the automated literature management tool EndNote. Another 41 articles were eliminated for not meeting inclusion criteria after review of the title and abstract, while the full texts of three articles were inaccessible. An additional 14 articles were eliminated after full text review for language, diagnosis, or microstate-related parameters. The exclusion reasons related to microstate parameters are as follows:① Insufficient microstate types: Articles that failed to identify or analyze at least four distinct microstate types (A, B, C, and D) or only described the global field power were excluded. This was to ensure a comprehensive comparison between individuals with ASD and the comparison group.② Inconsistent microstate definition: Articles that did not describe the directionality of microstates in text (e.g., microstate B: left frontal–right posterior), did not provide microstate scalp topography maps, or did not specify that their defined microstate images were consistent with those in previous studies were excluded.③ Lack of Relevant Data: Articles that did not provide sufficient data on the duration, frequency, or coverage of identified microstates were excluded.④ Methodological Issues: Studies that used non-resting-state EEG were excluded. Ultimately, seven articles were included in the meta-analysis (**Figure 1**).

### 3.2 Characteristics of included trials and participants

The seven studies in the meta-analysis enrolled a total of 194 ASD individuals, of which 133 were minors (66 young children and 67 children/adolescents), and 61 were adults ranging in age from 18.5 to 28.9 years. The total proportion of females was 13.9%, with one study (28) consisting entirely of males. Diagnostic criteria for ASD were relatively consistent across studies, with all individuals diagnosed by specialists according to DSM-IV, DSM-V, or ICD-10 criteria based on the Autism Diagnostic Observation Schedule (ADOS) (42) or Autism Diagnostic Interview (ADI) (43). In the seven included studies, 3/7 described the comparison group as typically developing, 2/7 described the comparison group as neurotypical, and 4/7 reported exclusion criteria indicating that the TD/NT groups excluded individuals with common psychiatric or developmental concerns beyond autism. Regarding intelligence, three studies partially described the methods and results of intelligence testing but did not provide specific data. One study did not provide any information on intelligence. The remaining studies described the tools used to measure intelligence and the specific IQ scores, as shown in **Table 1**.

**Table 1:** Summary of included studies.

Study	Sample size		Age		Sex		IQ (ASD/TD)	Diagnosis	Clustering method	Outcome	CE/OE
	ASD	comparison	ASD	comparison	ASD	comparison					
	(n = 194)	(n = 180)	(mean or range)	(mean or range)	(%females)	(%females)					
Jia and Yu (30)	15	18	11.6	8.9	0	0	(a)No Detail	ASD	T-AAHC	Duration, Frequency, Coverage	CE & OE
D’Croz-Baron et al. (28)	10	13	20–28	20–28	30%	61.50%	No Reported	ASD	k-means	Duration, Frequency, Coverage	CE
Nagabhushan et al. (31)	13	13	9.7	10.4	15.40%	15.40%	(b)101.3/102.1	ASD	T-AAHC	Duration, Frequency, Coverage	CE
Bochet et al. (29)	66	47	3.3	3.3	16.70%	17%	(c)73.4/110.4	ASD	k-means	Duration, Frequency, Coverage	CE
Takarae et al. (32)	39	48	12.19	11.68	8.61%	7.08%	(d) 106.11/109.64 105.06/106.15	ASD	k-means	Duration, Frequency	OE
Iftimovici et al. (25)	21	11	18.5	31	33.30%	45.50%	No Detail	ASD	k-means	Duration, Frequency, Coverage	CE
Das et al. (33)	30	30	28.91	29.33	33.30%	43.30%	(e)No Detail	ASD	k-means	Duration, Frequency, Coverage	CE

ASD, autism spectrum disorder; T-AAHC, agglomerate hierarchical clustering; k-means, k-means clustering; CE, eye closed; OE, eye opened.

(a): No significant difference in IQs between groups; all participant IQs > 66.

(b): Stanford Binet-5 Abbreviated IQ Test (SB5) ABIQ Standard Score: ASD 101.3 (19.8) TD 102.1 (9.9), p=0.13 (0.8974).

(c): Scales of Early Learning composite score (MSEL) Total DQ: ASD 73.4 (24.5, 55); TD: 110.4 (13.7, 35), p<0.0001.

(d): Wechsler Abbreviated Scale of Intelligence (WASI): VIQ 106.11 (18.00) 109.64 (11.75) t = 1.02, n.s. PIQ 105.06 (17.96) 106.15 (12.32) t < 1, n.s.

(e): All participants were “Intellectually able adults”.

Microstates were clustered into subtypes using either the T-AAHC or k-means clustering algorithm (44). All studies provided either the mean value and standard deviation of each outcome parameter, a data map to calculate the mean value and standard deviation (32), or the raw data to calculate the mean value and standard deviation (29) with the following exceptions (30) did not provide a mean coverage value for microstate D and takarae et al (32),. did not provide coverage data for any microstate. Most of these studies used resting-state closed-eye EEG data, but (30) analyzed mixed open-eye and closed-eye EEG data while (32) analyzed only open-eye EEG data.

### 3.3 Risk of bias

The clinical design and research methodologies of these studies were similar in most respects, although they appeared in a variety of journals with distinct emphases (neuroscience, psychiatry, brain topology, biomedical engineering). Four of these were classified as high-quality and three as moderate quality according to the AHRQ criteria for bias risk assessment. Five studies did not indicate the time period for individual recruitment, 5 did not explain individual exclusions from analysis, and five (non-population-based studies) did not clearly indicate whether participation was consensual. For detailed information on the risks of bias, refer to **Supplementary Table 3**.

### 3.4 Differences in microstate parameters between ASD and comparison groups

Results of the meta-analysis are summarized in **Table 2**. The mean duration of microstate B was longer in the ASD group than the comparison group (SMD = 0.83, 95%CI: 0.17–1.5), and the coverage of microstate B was greater in the ASD group (SMD = 0.54, 95%CI: 0.18–0.90). The occurrence frequency of microstate B was also greater in the ASD group, although the difference barely missed statistical significance (SMD = 0.45, 95%CI: 0.00–0.90). However, heterogeneity among studies reporting these metrics could not be excluded. In addition, the coverage of microstate C was lower in the ASD group than the comparison group (SMD = -0.61, 95%CI: -1.08 to -0.15), and heterogeneity among source studies was significant. There were no other significant differences in microstate parameters between groups.

**Table 2:** Meta-analysis results for microstate parameters.

Microstate	A	B	C	D
Duration(ms) SMD and 95% CI	0.08 (-0.52, 0.69)	0.83 (0.17, 1.50)	-0.27 (-0.95, 0.41)	-0.27 (-0.65, 0.11)
n	7	7	7	6
*p*	0.79	<0.05*	0.43	0.16
heterogeneity (*I*²)	0.86	0.88	0.89	0.63
Frequency SMD and 95% CI	-0.20 (-0.93, 0.52)	0.45 (0.00, 0.90)	-0.70 (-1.45, 0.05)	-0.55 (-1.91, -0.81)
n	7	7	7	6
*p*	0.59	0.05	0.07	0.43
heterogeneity (*I*²)	0.9	0.75	0.9	0.97
Coverage(%) SMD and 95% CI	-0.07 (-0.44, 0.30)	0.54 (0.18, 0.90)	-0.61 (-1.08, -0.15)	0.15 (-0.32, 0.61)
n	7	6	6	5
*p*	0.71	<0.01**	<0.05*	0.53
heterogeneity (*I*²)	0.52	0.49	0.68	0.64

Standardized Mean Difference, indicating the effect size.CI: Confidence Interval, added directly to the SMD cell in parentheses. n: Number of studies. *p*-value: Significance level, where * indicates *p* < 0.05 and ** indicates *p* < 0.01.*I*²: Heterogeneity index, representing the variability be.

### 3.5 Sensitivity analyses

**Figure 2:**
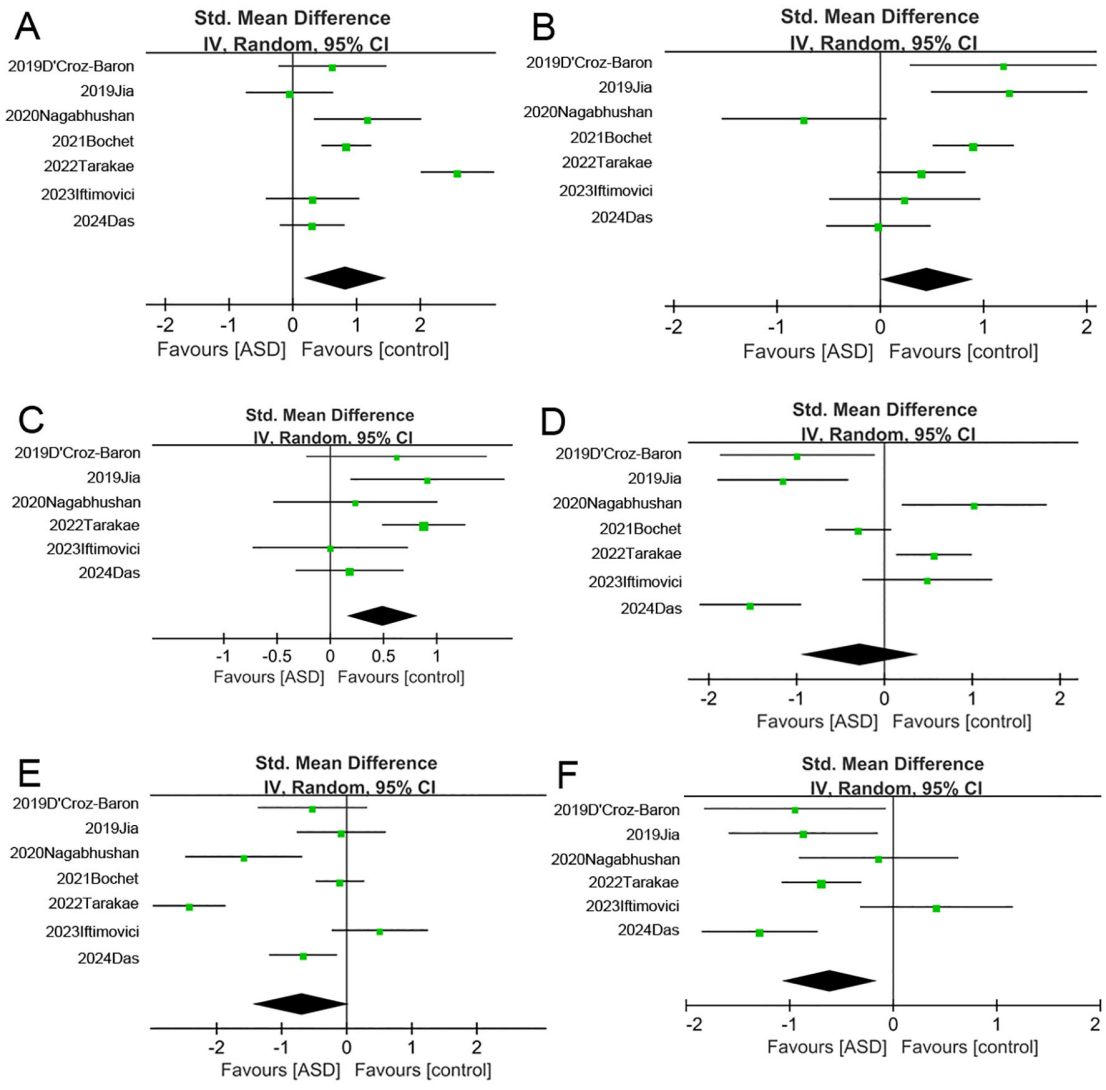
Forest map of meta-analysis. **(A–C)** The duration, frequency and coverage of Microstate **(B, D–F)** The duration, frequency and coverage of Microstate **(C)** As with **(A)**, **(C)**, and **(F)**, there are differences between the two groups in the duration and coverage of microstate B, as well as the coverage of microstate C.

**Figure 3:**
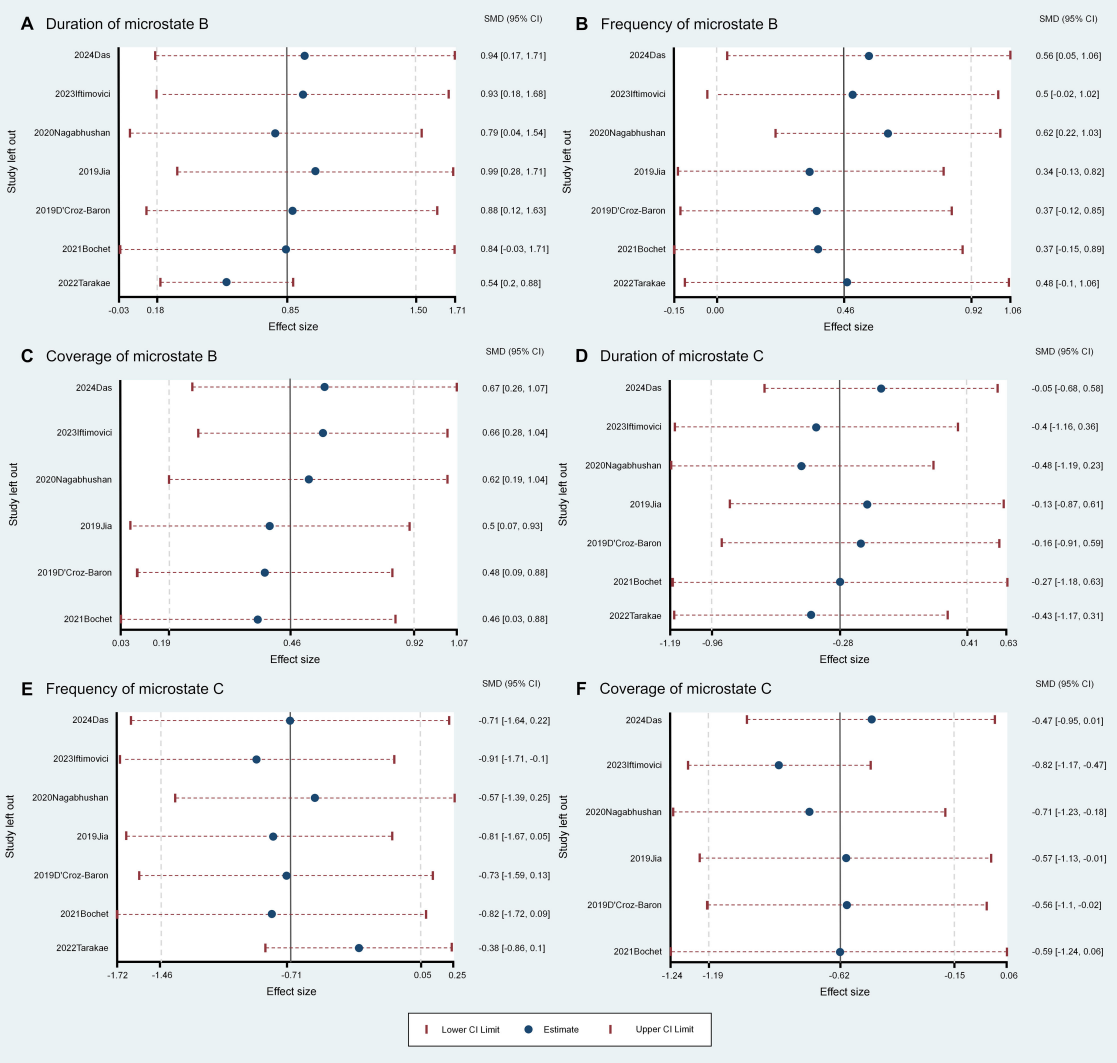
Leave-One-Out sensitivity analysis for the comparisons of microstate parameters between ASD and comparison groups. Panels A–C show the duration **(A)**, frequency **(B)**, and coverage **(C)** of Microstate B, with the coverage results of Microstate B remaining stable and unaffected by the removal of any single study **(C)**. Panels D–F display the duration **(D)**, frequency **(E)**, and coverage **(F)** of Microstate C.

The meta-analysis revealed several group differences in microstate B and C parameters, so we conducted further sensitivity analysis to assess the robustness and stability of these differences (**Figures 2**, **3**). The pooled group difference in microstate B coverage was not altered by removal of any individual source study, indicating that the meta-analysis result is stable. However, the pooled group difference in microstate B duration was substantially altered by removal of Bochet et al. (29) (ES=0.84, 95% CI=-0.02 to 1.71) and so cannot be considered robust. The group difference in microstate B frequency was altered by removal of several studies, indicating that the results are unstable. The nature of the pooled group difference in microstate C coverage was also altered by removal of several studies, especially Das et al. (33) and Iftimovici et al. (25), indicating that the results are unstable. Similarly, the group difference in microstate C occurrence was unstable. Finally, removal of individual studies did not alter the nature of the pooled group difference in microstate C duration but this difference was never significant.

### 3.6 Subgroup analysis

**Figure 4:**
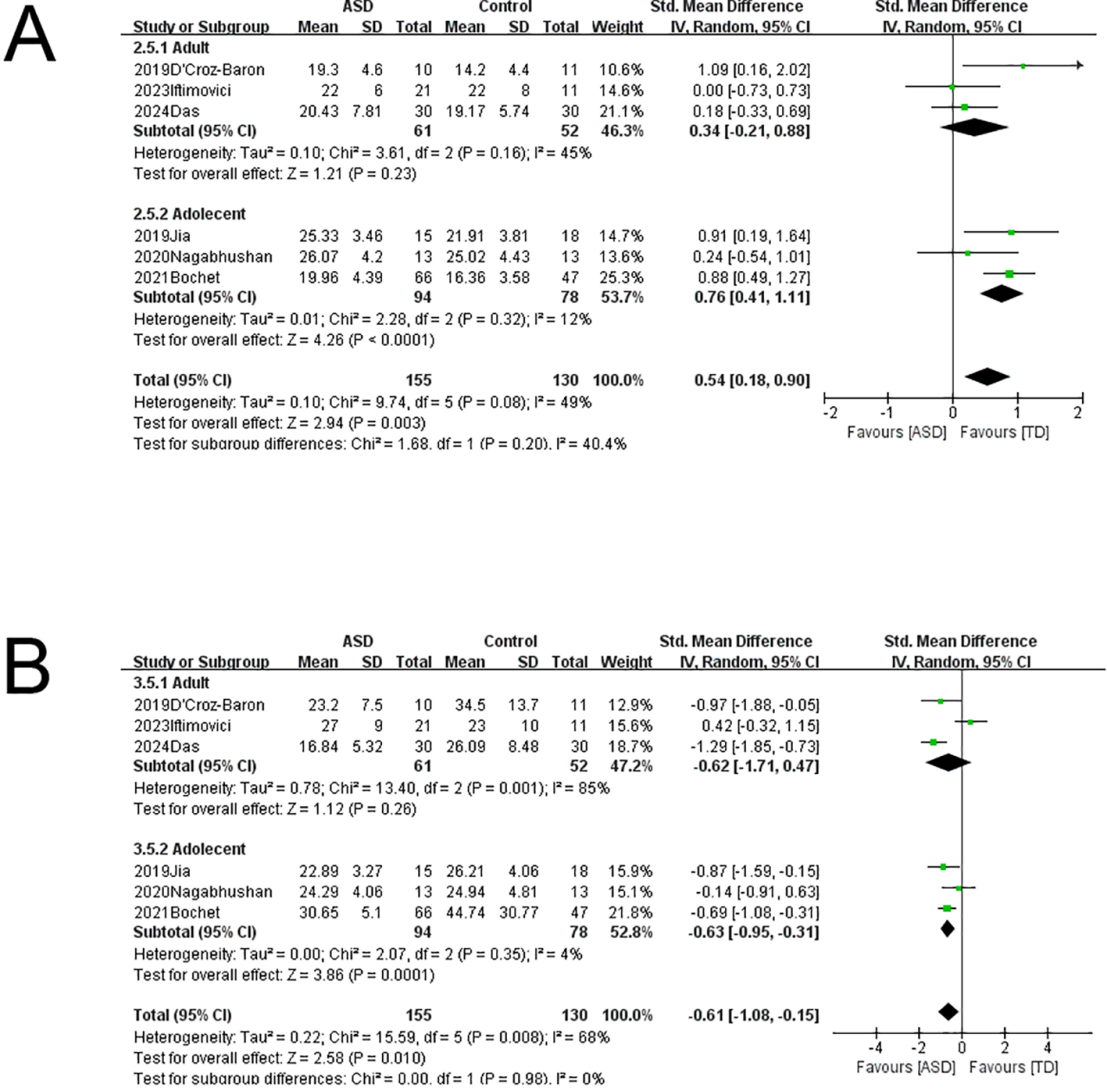
Subgroup analysis of EEG microstates. **(A)** Microstate B coverage was significantly higher in children and adolescents with ASD compared to the age-matched comparison group, and heterogeneity could be excluded. **(B)** Microstate C coverage was significantly lower in children and adolescents with ASD compared to the age-matched comparison group, and heterogeneity could be excluded.

To explore the sources of heterogeneity, we conducted subgroup analyses with stratification by age (children/adolescents vs. adults) and microstate-based clustering method (k-means vs. T-AAHC) (**Figure 4**). There were no significant changes in microstate A parameters whether stratified by age or clustering method. However, microstate B coverage was significantly greater in children/adolescents of the ASD group compared to the comparison group without significant heterogeneity (SMD = 0.76, 95%CI: 0.41–1.11, I² = 12%), but coverage did not differ between ASD and comparison adults. Therefore, age may be a significant source of heterogeneity in microstate B coverage. Microstate C coverage was also significantly greater among children/adolescents of the ASD group compared to the comparison group without significant heterogeneity (SMD = -0.63, 95%CI: -0.95 to -0.31, I² = 4%) but did not differ between ASD and comparison adults. Therefore, age may also be a source of heterogeneity for microstate C coverage. All other changes in microstate B and C parameters due to subgroup stratification demonstrated substantial heterogeneity. Finally, the heterogeneity of microstate D parameters were also reduced by age stratification, although there were no significant differences between ASD and comparison groups.

### 3.7 Publication bias

**Figure 5:**
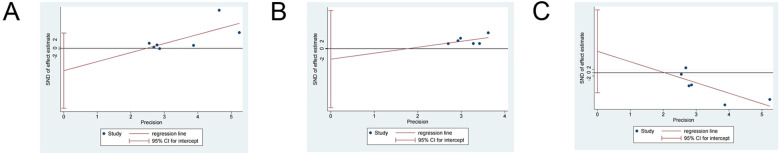
The results of Egger’s test for publication bias. The null hypothesis of no publication bias cannot be rejected when the 95% CI intercept includes 0. According to Egger’s test, there is no evidence for publication bias among source studies for **(A)** microstate B duration, **(B)** microstate B coverage, or **(C)** microstate C coverage.

As shown in **Figure 5**, Egger’s tests revealed no publication bias among studies reporting microstate B duration (t=-1.52, p=0.188), microstate B coverage (t=-0.6, p=0.581), or microstate C coverage (t=1.43, p=0.225).

## 4 Discussion

This meta-analysis revealed multiple EEG microstate abnormalities in individuals with ASD, including lower microstate C coverage, higher microstate B coverage, and longer microstate B duration compared to comparisons. Our main results are consistent with the previous systematic review (34) and have been consolidated through quantitative research methods. Many studies (17, 21, 45, 46) investigating the relationship between EEG microstates and resting-state fMRI have found that Microstate B is associated with the DMN and the central executive network (CEN), while Microstate C is related to the Visual Network (VIS). Abnormalities in these networks may be closely related to the occurrence and development of ASD (13–15). However, heterogeneity could not be excluded or was significant across studies included in these pooled comparisons. In fact, only the difference in microstate B coverage was robust according to sensitivity analysis. Subgroup analysis further identified age as the main cause of heterogeneity, while the clustering method used had little effect and there was no detectable influence of publication bias.

Our finding that age is the main source of heterogeneity in microstate B and C time parameters is broadly consistent with previous studies of healthy populations. For example (47), found that microstate C duration increased with age in comparison females ranging from 6 to 87 years old and that microstate D frequency increased with age in comparison males of the same age range. Similarly, takarae et al. (32) found that microstate C duration was positively correlated with age among comparison children. In the current meta-analysis, microstate C coverage gradually increased with age, but this increase was not uniform, resulting in substantial heterogeneity among studies. Age also influences EEG microstate time parameters in addition to microstate B and C duration. For example, Bagdasarov et al. (48) found reduced microstate 4 (D) duration, frequency, and coverage with age among male children, while Hill (49) found associations of age with microstate E duration and occurrence frequency among female children 4–12 years old. Therefore, various microstate time parameters are differentially influenced by age, leading to marked results heterogeneity across studies.

These EEG microstates are associated with distinct neural activity patterns in resting-state functional networks (RSNs) (21, 50), suggesting that abnormalities in microstate features can reflect network-level disorganization underlying the condition. Watanabe et al. (51) reported that while comparison brain activity usually transitioned between major states through stable intermediate states, ASD individuals exhibited fewer neural state transitions due to intermediate state instability. In turn, an unstable intermediate state could significantly alter microstate duration, frequency of occurrence, and coverage. These abnormalities in network activity may also arise from changes in connectivity strength among network nodes. For instance, Di Martino et al. (52) found both network hypoconnectivity and hyperconnectivity in a large sample of ASD individuals (n = 1112) ranging in age from 7 to 64 years, including weaker connectivity within the default mode network (DMN). It was posited that these abnormalities could lead to intermediate state instability, thereby disrupting normal brain state transitions. Haghighat et al. (53) reported a transition from general hyperconnectivity to mixed hyper- and hypoconnectivity with age in ASD. This change in connectivity may be the key to atypical development of brain networks and changes in microstate with age. In addition, abnormalities in myelination (54) and synaptic pruning (55, 56) during maturation may indirectly influence network structure and microstate patterns.

However, the studies included in this meta-analysis exhibited variability in IQ data. In this study (32), the average IQ of the ASD group was 105.5, which is significantly higher than that of the general ASD population. This may limit the generalizability of the results. Since IQ is associated with EEG activity, as demonstrated by Agcaoglu et al. (57) who found that the lateralization of resting-state networks is significantly correlated with IQ, it suggests that IQ may directly influence microstate characteristics. Therefore, there may be differences in EEG microstate features between high-functioning and low-functioning individuals with ASD. Additionally, the DSM-5 requires cognitive or developmental assessments for the diagnosis of ASD. However, four studies (25, 28, 30, 34) did not report IQ data in detail, which may mask the impact of cognitive abilities on EEG patterns and affect the rigor of sample representation. Therefore, future studies should strictly adhere to diagnostic criteria, report IQ data in detail, and use standardized assessment tools or conduct stratified analyses for ASD groups with different functional levels to enhance the transparency, reliability, and generalizability of the results.

### 4.1 Limitations

This study has several limitations, most notably the small number of studies included and the relatively small sample sizes in individual studies, which reduced statistical power and prevented certain subgroup analyses. Further, three of the seven studies were of only moderate quality according to risk of bias assessment. Future studies on the EEG microstate characteristics of ASD must adhere more closely to reporting guidelines for reduced bias risk and recruit larger individual samples within narrower age ranges. Further, future longitudinal studies are essential to assess the age-dependence of EEG microstate abnormalities associated with ASD.

## 5 Conclusion

This meta-analysis reveals abnormalities in EEG microstate duration, coverage, and occurrence frequency among ASD individuals, including an increase in microstate B coverage that was relatively reproducible across studies. However, many other differences from control study participants were inconsistent across studies, and we further show that age is a major source of this heterogeneity. Future studies with expanded sample sizes within more restricted age ranges and long-term follow-up are essential to clarify the clinical utility of EEG microstate abnormalities for ASD diagnosis and monitoring.

## Data Availability

The original contributions presented in the study are included in the article/**Supplementary Material**. Further inquiries can be directed to the corresponding authors.
