# Supplementary material for: Distinct features of EEG microstates in autism spectrum disorder revealed by meta-analysis: the contribution of individual age to heterogeneity across studies
Source: Front Psychiatry. 2025 Apr 22;16:1531694. doi: 10.3389/fpsyt.2025.1531694 (PMC12052564; doi:10.3389/fpsyt.2025.1531694)
Supplement: Supplementary file 2 [file Table2.docx]

Supplement table 2. Retrieval strategy

| PubMed | |
| --- | --- |
| #4 | #1 and #2 and #3 |
| #3 | (microstate[Title/Abstract]) |
| #2 | ((((Electroencephalography[MeSH Major Topic])) OR (Electroencephalography[Title/Abstract])) OR (Electroencephalography[Title/Abstract])) OR (EEG[Title/Abstract])) |
| #1 | (((((ADHD[MeSH Terms])) OR (ADHD[Title/Abstract])) OR (Attention Deficit Hyperactivity Disorder[Title/Abstract])) OR (Attention Deficit Disorder[Title/Abstract])) OR (Hyperactivity[Title/Abstract]) |
| Web of Science | |
| #4 | #1 and #2 and #3 |
| #3 | TS=(microstate) |
| #2 | (TS=(electroencephalogram)) OR TS=(EEG) |
| #1 | (((TS=(Autism Spectrum Disorder)) OR TS=(ASD)) OR TS=(Autistic disorder)) OR TS=(Autism) |
| EBSCO and PsychInfo | |
| S4 | S1 and S2 and S3 |
| S3 | AB microstate |
| S2 | AB OR AB EEG |
| S1 | AB Autism Spectrum Disorder OR AB Autistic Disorder OR AB ASD |
| Cochrane Library | |
| #4 | #1 and #2 and #3 |
| #1 | (Autism Spectrum Disorder):ti,ab,kw OR (ASD):ti,ab,kw OR (Disorder, Autistic Spectrum):ti,ab,kw OR (Autistic Disorder):ti,ab,kw OR (Autism) |
| #2 | (EEG):ti,ab,kw; OR AB EEG |
| #3 | (microstate):ti,ab,kw |
