# Supplementary material for: Distinct features of EEG microstates in autism spectrum disorder revealed by meta-analysis: the contribution of individual age to heterogeneity across studies
Source: Front Psychiatry. 2025 Apr 22;16:1531694. doi: 10.3389/fpsyt.2025.1531694 (PMC12052564; doi:10.3389/fpsyt.2025.1531694)
Supplement: Supplementary file 3 [file Table3.docx]

Supplementary Table 3. Methodological quality appraisal results based on the AHRQ tool for each study.

|  | Item 1 | Item 2 | Item 3 | Item 4 | Item 5 | Item 6 | Item 7 | Item 8 | Item 9 | Item 10 | Item 11 | Total score | Quality |
| --- | --- | --- | --- | --- | --- | --- | --- | --- | --- | --- | --- | --- | --- |
| 2024Das | Y | Y | N | U | Y | Y | Y | Y | Y | Y | U | 8 | H |
| 2023Iftimovici | Y | Y | N | Y | Y | Y | N | Y | Y | Y | U | 8 | H |
| 2022Tarakae | Y | Y | Y | Y | Y | Y | Y | Y | Y | Y | U | 10 | H |
| 2021Bochet | Y | Y | Y | U | Y | Y | N | Y | Y | Y | U | 8 | H |
| 2020Nagabhushan | Y | Y | N | U | Y | Y | N | Y | Y | Y | U | 7 | M |
| 2019D'Croz-Baron | Y | Y | N | U | Y | Y | N | Y | Y | Y | U | 7 | M |
| 2019Jia | Y | Y | N | U | Y | Y | N | Y | Y | Y | U | 7 | M |
| *Note: Y, yes; N, no; U, unclear; H, high quality; M, medium quality.* | | | | | | | | | | | | | |
| Item 1: Define the source of information (survey, record review). | | | | | | | | | | | | | |
| Item 2: List inclusion and exclusion criteria for exposed and unexposed subjects (cases and controls) or refer to previous publications. | | | | | | | | | | | | | |
| Item 3: Indicate time period used for identifying patients. | | | | | | | | | | | | | |
| Item 4: Indicate whether or not subjects were consecutive if not population-based. | | | | | | | | | | | | | |
| Item 5: Indicate if evaluators of subjective components of study were masked to other aspects of the status of the participants. | | | | | | | | | | | | | |
| Item 6: Describe any assessments undertaken for quality assurance purposes (e.g., test/retest of primary outcome measurements). | | | | | | | | | | | | | |
| Item 7: Explain any patient exclusions from analysis. | | | | | | | | | | | | | |
| Item 8: Describe how confounding was assessed and/or controlled. | | | | | | | | | | | | | |
| Item 9: If applicable, explain how missing data were handled in the analysis. | | | | | | | | | | | | | |
| Item 10: Summarize patient response rates and completeness of data collection. | | | | | | | | | | | | | |
| Item 11: Clarify what follow-up, if any, was expected and the percentage of patients for which incomplete data or follow-up was obtained. | | | | | | | | | | | | | |
