# Supplementary material for: Distinct features of EEG microstates in autism spectrum disorder revealed by meta-analysis: the contribution of individual age to heterogeneity across studies
Source: Front Psychiatry. 2025 Apr 22;16:1531694. doi: 10.3389/fpsyt.2025.1531694 (PMC12052564; doi:10.3389/fpsyt.2025.1531694)
Supplement: Supplementary file 4 [file Table4.docx]

**Supplementary table 4** Data of microstate parameters included in the meta-analysis.

|  |  | Duration（ms） | | | | | | Frequency of occurance | | | | | | Coverage（%） | | | | | |
| --- | --- | --- | --- | --- | --- | --- | --- | --- | --- | --- | --- | --- | --- | --- | --- | --- | --- | --- | --- |
|  |  | ASD | | | ctrl | | | ASD | | | ctrl | | | ASD | | | ctrl | | |
|  |  | Mean | SD | n | Mean | SD | n | Mean | SD | n | Mean | SD | n | Mean | SD | n | Mean | SD | n |
| Microstate A | 2024Das | 81.88 | 10.07 | 30 | 84.61 | 11.66 | 30 | 2.675 | 0.3649 | 30 | 2.227 | 0.4954 | 30 | 22.08 | 4.96 | 30 | 19 | 5.59 | 30 |
|  | 2023Iftimovici | 89 | 9 | 21 | 85 | 25 | 11 | 1.9 | 0.4 | 21 | 1.7 | 0.6 | 11 | 17.00 | 4.00 | 21 | 16 | 6 | 11 |
|  | 2020Nagabhushan | 81.32 | 12.75 | 13 | 73.58 | 11.53 | 13 | 3.39 | 0.67 | 13 | 3.87 | 0.41 | 13 | 24.84 | 4.85 | 13 | 26.21 | 3.97 | 13 |
|  | 2019Jia | 71.855 | 7.64 | 15 | 79.57 | 6.25 | 18 | 3.61 | 0.54 | 15 | 3.56 | 0.49 | 18 | 23.86 | 3.12 | 15 | 25.84 | 3.27 | 18 |
|  | 2019D'Croz-Baron | 76.29 | 6.08 | 10 | 78.79 | 6.54 | 13 | 1.73 | 0.36 | 10 | 1.71 | 0.43 | 13 | 15.20 | 4.30 | 10 | 15.7 | 5 | 13 |
|  | 2021Bochet | 71.58 | 4.8 | 66 | 71.91 | 3.99 | 47 | 2.36 | 0.44 | 66 | 2.5 | 0.29 | 47 | 19.40 | 4.85 | 66 | 20.65 | 3.51 | 47 |
|  | 2022Takerae | 63.08 | 0.71 | 39 | 61.89 | 0.95 | 48 | 1.38 | 0.04 | 39 | 1.45 | 0.04 | 48 |  |  |  |  |  |  |
| Microstate B | 2024Das | 86.83 | 18.48 | 30 | 81.76 | 14.29 | 30 | 2.307 | 0.5602 | 30 | 2.316 | 0.4153 | 30 | 20.43 | 7.81 | 30 | 19.17 | 5.74 | 30 |
|  | 2023Iftimovici | 99 | 12 | 21 | 93 | 28 | 11 | 2.2 | 0.5 | 21 | 2.1 | 0.1 | 11 | 22.00 | 6.00 | 21 | 22 | 8 | 11 |
|  | 2020Nagabhushan | 83.37 | 13.08 | 13 | 69.32 | 9.91 | 13 | 3.39 | 0.64 | 13 | 3.87 | 0.62 | 13 | 26.07 | 4.20 | 13 | 25.02 | 4.43 | 13 |
|  | 2019Jia | 73.76 | 9.39 | 15 | 74.2 | 7.26 | 18 | 3.74 | 0.36 | 15 | 3.19 | 0.48 | 18 | 25.33 | 3.46 | 15 | 21.91 | 3.81 | 18 |
|  | 2019D'Croz-Baron | 80.6 | 4.45 | 10 | 76.4 | 7.7 | 13 | 2.1 | 0.41 | 10 | 1.6 | 0.4 | 13 | 19.60 | 4.60 | 10 | 14.2 | 4.4 | 13 |
|  | 2021Bochet | 72.48 | 4.75 | 66 | 68.86 | 3.5 | 47 | 2.40 | 0.35 | 66 | 2.09 | 0.33 | 47 | 19.96 | 4.39 | 66 | 16.36 | 3.58 | 47 |
|  | 2022Takerae | 65.68 | 0.71 | 39 | 63.08 | 1.18 | 48 | 1.76 | 0.05 | 39 | 1.73 | 0.09 | 48 |  |  |  |  |  |  |
| Microstate C | 2024Das | 74.97 | 7.59 | 30 | 101.94 | 23.45 | 30 | 2.209 | 0.4834 | 30 | 2.522 | 0.4374 | 30 | 16.84 | 5.32 | 30 | 26.09 | 8.48 | 30 |
|  | 2023Iftimovici | 109 | 21 | 21 | 97 | 29 | 11 | 2.4 | 0.5 | 21 | 2.1 | 0.7 | 11 | 27.00 | 9.00 | 21 | 23 | 10 | 11 |
|  | 2020Nagabhushan | 81.56 | 16.67 | 13 | 67.32 | 9.27 | 13 | 3.24 | 0.42 | 13 | 4.04 | 0.55 | 13 | 24.29 | 4.06 | 13 | 24.94 | 4.81 | 13 |
|  | 2019Jia | 71.75 | 9.17 | 15 | 82.01 | 8.2 | 18 | 3.46 | 0.5 | 15 | 3.5 | 0.44 | 18 | 22.89 | 3.27 | 15 | 26.21 | 4.06 | 18 |
|  | 2019D'Croz-Baron | 87.16 | 8.67 | 10 | 103.35 | 19.4 | 13 | 2.24 | 0.47 | 10 | 2.54 | 0.6 | 13 | 23.20 | 7.50 | 10 | 34.5 | 13.7 | 13 |
|  | 2021Bochet | 84.12 | 5.81 | 66 | 100.37 | 83.87 | 47 | 3.08 | 0.3 | 66 | 3.11 | 0.26 | 47 | 30.65 | 5.10 | 66 | 44.74 | 30.77 | 47 |
|  | 2022Takerae | 71.36 | 0.95 | 39 | 70.89 | 0.71 | 48 | 2.26 | 0.28 | 39 | 2.74 | 0.08 | 48 |  |  |  |  |  |  |
| Microstate D | 2024Das | 77.13 | 7.45 | 30 | 79.99 | 10.11 | 30 | 2.395 | 0.4657 | 30 | 1.838 | 0.4589 | 30 | 18.70 | 5.19 | 30 | 14.85 | 4.56 | 30 |
|  | 2023Iftimovici | 88 | 13 | 21 | 91 | 28 | 11 | 1.7 | 0.4 | 21 | 2.3 | 0.6 | 11 | 16.00 | 5.00 | 21 | 21 | 10 | 11 |
|  | 2020Nagabhushan | 63.99 | 11.1 | 13 | 80.67 | 15.32 | 13 | 3.46 | 0.51 | 13 | 4.04 | 0.58 | 13 | 24.79 | 4.31 | 13 | 23.82 | 4.12 | 13 |
|  | 2019D'Croz-Baron | 77.59 | 6.94 | 10 | 74.71 | 11.76 | 13 | 1.81 | 0.38 | 10 | 1.5 | 0.64 | 13 | 16.20 | 4.60 | 10 | 13.6 | 8.3 | 13 |
|  | 2021Bochet | 70.01 | 4.17 | 66 | 69.3 | 4.18 | 47 | 1.95 | 0.46 | 66 | 1.97 | 0.44 | 47 | 15.57 | 4.35 | 66 | 15.61 | 4.59 | 47 |
|  | 2022Takerae | 62.37 | 0.95 | 39 | 62.84 | 0.71 | 48 | 1.67 | 0.06 | 39 | 1.83 | 0.05 | 48 |  |  |  |  |  |  |
